# Enhanced Hydrophilicity and Biocompatibility of Dental Zirconia Ceramics by Oxygen Plasma Treatment

**DOI:** 10.3390/ma8020684

**Published:** 2015-02-16

**Authors:** Ching-Chou Wu, Chung-Kai Wei, Chia-Che Ho, Shinn-Jyh Ding

**Affiliations:** 1 Department of Bio-Industrial Mechatronics Engineering, National Chung Hsing University, Taichung City 402, Taiwan; E-Mail: ccwu@dragon.nchu.edu.tw; 2 Institute of Oral Science, Chung Shan Medical University, Taichung City 402, Taiwan; E-Mails: tiyeg2@gmail.com (C.-K.W.); sfox1223@gmail.com (C.-C.H.); 3 Department of Dentistry, Chung Shan Medical University Hospital, Taichung City 402, Taiwan

**Keywords:** zirconia, all ceramic, oxygen plasma, hydrophilicity, biocompatibility

## Abstract

Surface properties play a critical role in influencing cell responses to a biomaterial. The objectives of this study were (1) to characterize changes in surface properties of zirconia (ZrO_2_) ceramic after oxygen plasma treatment; and (2) to determine the effect of such changes on biological responses of human osteoblast-like cells (MG63). The results indicated that the surface morphology was not changed by oxygen plasma treatment. In contrast, oxygen plasma treatment to ZrO_2_ not only resulted in an increase in hydrophilicity, but also it retained surface hydrophilicity after 5-min treatment time. More importantly, surface properties of ZrO_2_ modified by oxygen plasma treatment were beneficial for cell growth, whereas the surface roughness of the materials did not have a significant efficacy. It is concluded that oxygen plasma treatment was certified to be effective in modifying the surface state of ZrO_2_ and has the potential in the creation and maintenance of hydrophilic surfaces and the enhancement of cell proliferation and differentiation.

## 1. Introduction

Due to the merits of zirconia, such as high mechanical strength and toughness, and good abrasion resistance and chemical stability *in vivo*, it is currently used in the femoral head of hip prostheses and dental restoration [1,2]. However, this material has no direct bone bonding properties or osteoconduction behavior, except it shows a morphological fixation with the surrounding tissues alone [1,2,3]. To pursue the high bioactivity is still a concerned theme, although commercial ZrO_2_ systems are available today.

Given that biological tissues interact with only the outermost atomic layers of an implant, a number of surface modification methods to improve inert properties of the ZrO_2_ surfaces in an effort of enhancing cell-material interaction have been introduced. They included roughening and acid-etching [4], micro-arc oxidation [5,6], bioactive coating [7,8], laser modification [9] and plasma treatment [10,11]. For example, Pelaez-Vargas *et al.* used a combined approach consisting of sol–gel technology and soft lithography to coat isotropic micro-patterned silica coatings on zirconia substrates [8]. Compared with the complex methods of coating, oxygen plasma treatment is relatively inexpensive and time efficient, as it is simple to transform the hydrophobic hydrocarbon surfaces of ZrO_2_ to the hydrophilic surfaces. Plasma treatment has been used to modify the surface physicochemical properties of polymers, glasses, ceramics or metals [4,10,11,12]. In a previous study [12], we found that the water contact angle of polydimethylsiloxane surfaces varied from 103° (hydrophobicity) to approximately 10° (hydrophilicity) after oxygen plasma treatment. A recent study indicated that the application of cold plasma may be supportive in the treatment of peri-implant lesions and improve the process of re-osseointegration of titanium implants [10]. Yoshinari and co-workers found that blast/acid etching, oxygen plasma treatment and ultraviolet light irradiation greatly increased the surface wettability of the zirconia disks, resulting in superhydrophilicity [13]. Shon *et al.* indicated that helium plasma treatments on powder-injection molded zirconia implants made the surface more hydrophilic and enhanced the osseointegration of the implants in a rabbit tibiae mode without changing the microtopography [14].

In this study, attempts have been made to create a hydrophilic layer on the commercially available dental ZrO_2_ surfaces with either polishing or sandblasting pretreatment followed by oxygen plasma irradiation for achieving more desirable cell responses. Evaluation of this potential method included morphology and phase composition, particularly for cell responses to oxygen plasma-treated ZrO_2_ surfaces. In addition, the water contact angle of the treated ZrO_2_ surfaces was monitored with respect to time to monitor the stability. More importantly, MG63 human osteoblast-like cells were used to evaluate effect of plasma treatment on cell behavior before *in vivo* study.

## 2. Results and Discussion

### 2.1. Morphology

**Figure 1:**
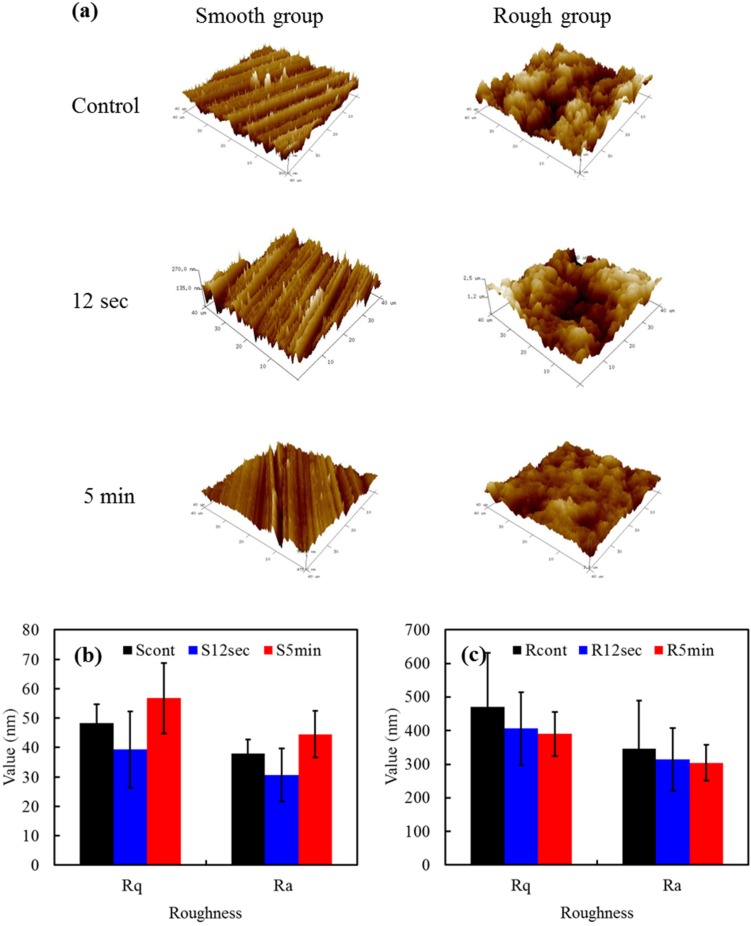
(**a**) Atomic Force Microscope (AFM) images of various samples; The roughness values in Rq and Ra of (**b**) smooth samples; and (**c**) rough samples with and without the plasma treatment.

Figure 1a shows the Atomic Force Microscope (AFM) images of the smooth and rough samples before and after oxygen plasma treatment. Before plasma treatment, the smooth sample presented longitudinal, parallel grooves while the rough sample resulted in a highly irregular structure. Not surprisingly, the surface morphology was does not altered by oxygen plasma treatment, in agreement with previous studies [11,14], possibly because the used power of plasma was not enough to change the morphology of hard ZrO_2_ surface. The roughness of the TZP samples after exposure to the plasma was also investigated. No significant roughness values (*p* > 0.05) in Ra (or Rq) were observed for smooth samples (30–40 nm) or rough samples (300–350 nm) (Figure 1b,c) when 12 s or 5 min irradiation duration was applied. It seems reasonable to suspect that the polished surfaces had significantly (*p* < 0.05) lower roughness values than the sandblasted samples.

### 2.2. Phase Composition

**Figure 2:**
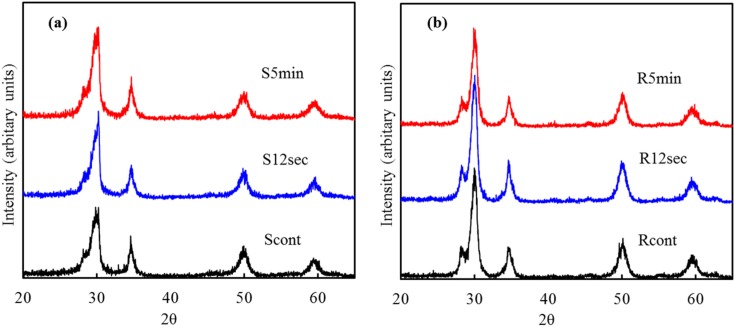
Low angle XRD patterns of (**a**) smooth; and (**b**) rough sample surfaces subjected to different oxygen plasma treatment for 0 s (the control), 12 s and 5 min.

Figure 2 shows the low angle XRD patterns of smooth and rough sample surfaces before and after O_2_ plasma treatment for different time duration. The sample was composed of tetragonal zirconia phase (t-ZrO_2_) with a trace of monoclinic zirconia (m-ZrO_2_). Three characteristic peaks located at around 30.2°, 34.6° and 49.9° can be attributed to (101), (110) and (112) tetragonal crystal faces of YZP [15]. A significant peak of (−111) monoclinic crystals at 28.2° was detected; the (200) and (220) monoclinic crystals overlapped with the (110) and (112) tetragonal phases, respectively [15]. The oxygen plasma did not induce any significant phase evolution, in concurrence with the results of the surface morphology.

### 2.3. XPS Analysis

**Figure 3:**
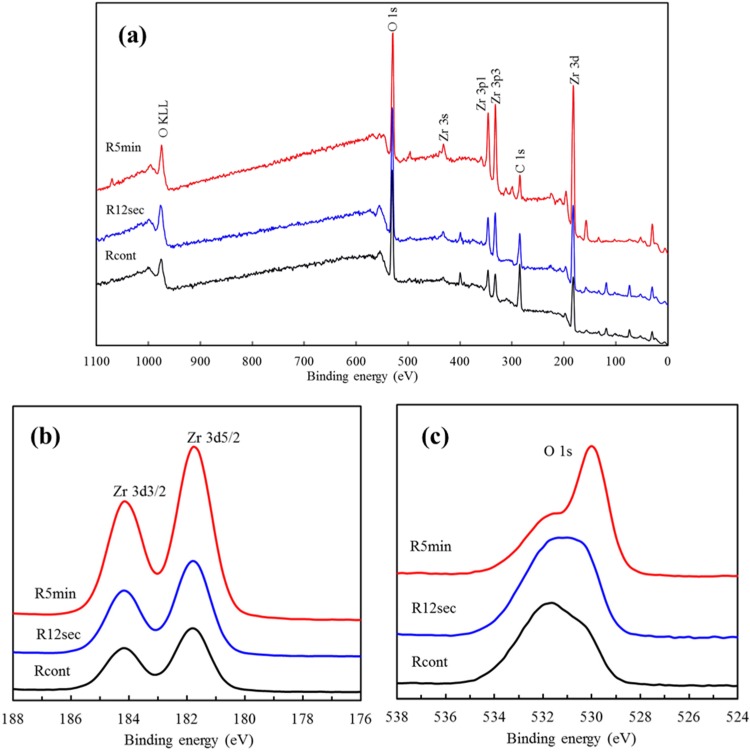
(**a**) XPS spectra survey of the rough sample surfaces with and without the plasma treatment; (**b**) high-resolution XPS analysis of Zr 3d; (**c**) high-resolution XPS analysis of O 1s.

Figure 3a shows the XPS spectrum of the rough sample surfaces before and after oxygen plasma treatment taken in the range from 0 to 1100 eV. Plasma-treated TZP samples had a lower C/O atomic ratio (0.36) when compared with that of the TZP control (0.46). It is considered that the carbon is caused by the impurity-contained substrates and the air contamination such as CO_2_ and CO molecules [16]. The excited particles created in plasma react with the surface layer of hydrocarbons and carbonate molecules oxidizing them to form water and carbon dioxide, which are desorbed from the surface and pumped away [17]. This might explain why a lower C/O ratio was found on the plasma-treated surfaces.

The high-resolution XPS spectra of the Zr 3d peak for the rough YZP samples with and without plasma treatment are shown in Figure 3b. The binding energies of the core levels were calibrated using the binding energy of C 1s = 284.8 eV. The binding energies of Zr 3d3/2 and Zr 3d5/2 were around 184.2 eV and 181.8 eV [6], which clearly indicated the presence of fully oxidized zirconium in its Zr^4+^ state, as expected in ZrO_2_ [18,19]. It is noteworthy that the intensity of Zr 3d on the plasma-treated surface appeared to be somewhat increased and shifted to lower bonding energy compared with the control, possibly due to refinement of the microstructure.

This high-resolution Zr spectrum did not allow for separation of the constitution of zirconium hydroxide and ZrO_2_ in the Zr 3d signal. Hence, the O 1s core-level spectra were assayed to gain further information. The differences in the bonding energy level of the O 1s peak between the pristine and plasma-treated YZP can be clearly seen, as shown in Figure 3c. The O 1s spectra consisted of three peaks originating from oxygen in the Zr-O bond at 530.0 eV, and oxygen in hydroxyl group (zirconium hydroxide and H_2_O) at 531.0 eV and 531.8 eV [4,6]. The plasma treatment induced the creation of ZrO_2_ oxide layer instead of zirconium hydroxide layer, and this effect was more pronounced at the extended treatment time.

### 2.4. Contact Angle

**Figure 4:**
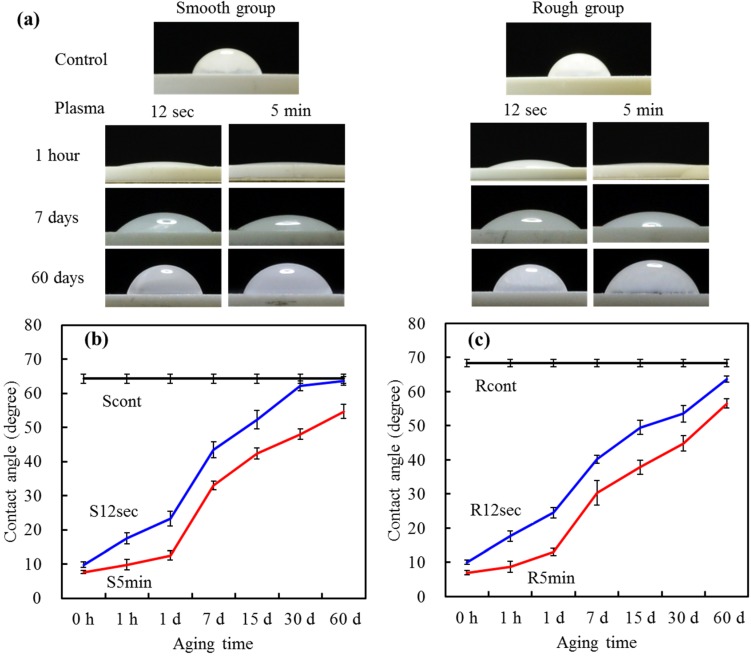
(**a**) Images of water droplets on the different sample surfaces before and after aging for different time points; the changes in the water contact angle of (**b**) smooth samples; and (**c**) rough samples with and without the plasma treatment after different aging time-points.

Water contact angle is used to measure surface hydrophilicity by evaluating how much a water droplet could spread on a surface. Figure 4a shows pictures of contact angle measurement of YZP samples that were oxygen plasma-treated for 0, 12 s and 5 min, which followed by different aging time-points. The YZP control surface had a large water contact angle of approximately 65°, which was close to the published data in other studies [4].

To reduce the contact angle, we conducted oxygen plasma treatment to the surfaces. Regardless of the exposure time of oxygen plasma, the contact angle measured was less than 10°, denoting a good hydrophilic surface property. The lower the contact angle, the more hydrophilic the surface was. The oxygen plasma can improve effectively the hydrophilicity of the YZP surfaces, possibly due to elimination of adsorbed hydrocarbon and carbonate molecules, as evidenced by XPS results. As a surface becomes more oxidized, or has more ionizable groups introduced to it, hydrogen bonding with the water becomes more facile and the droplet spreads along the hydrophilic surface, resulting in a lower contact angle [20,21,22]. In addition, the wettability of the samples strongly depends on the surface cleaning [23].

Aging of the plasma-treated surfaces was studied by measuring the time dependence of the contact angle. Durability of surface hydrophilicity is essential for clinical applications. At 7 days of aging, the average contact angles for S12 s, S5 min, R12 s and R5 min groups were 44°, 33°, 40° and 30°, respectively, significantly lower (*p* < 0.05) than that of the control (around 65°). More importantly, the 5-min treatment groups had significantly (*p* < 0.05) better hydrophilicity than the 12-s treatment groups throughout the aging time, regardless of the pretreatment conditions (Figure 4b,c). After 60 days of aging time, the water contact angle on the 12-s-treated surfaces was close to that of the control, whereas 5-min-treated surfaces remained in the range of 55°–57°. This can be explained by the fact that the longer treatment time allowed for the modification to take place at a thicker depth. Oxygen plasma treatment always assures a high surface energy, which in turn facilitates surface migration of atoms [17]. Nevertheless, the plasma-treated surface gradually recovered the original hydrophobicity with time, as reported in an earlier article [12]. This phenomenon is called “hydrophobic recovery” possibly due to the reorientation of crystal structure or adsorption of hydrocarbons at the surface. In addition to chemical composition, hydrophilicity of materials might depend on the surface roughness [24]. However, the pretreatment conditions (polishing and sandblasting) did not influence noticeably the water contact angle. The compromise between roughness and morphology may be the reason.

### 2.5. Cell Attachment

**Figure 5:**
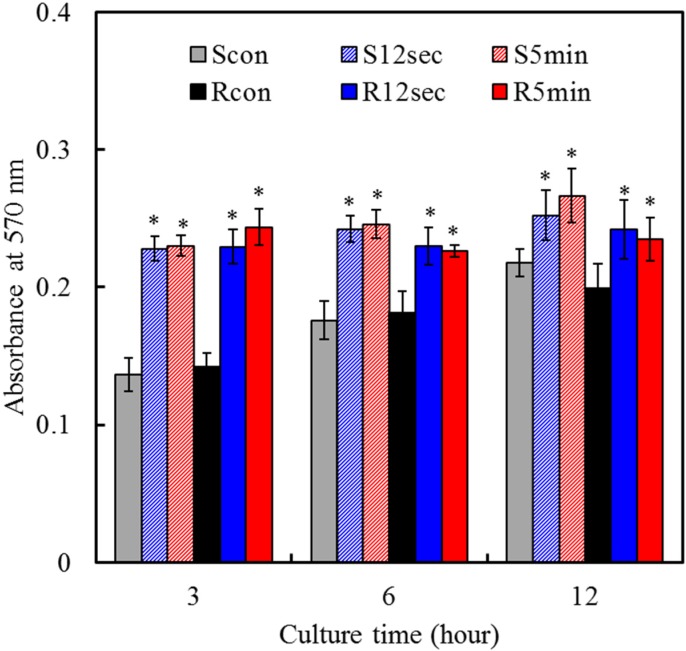
MTT assay for MG63 cells cultured on various samples to reveal cell attachment at various time points. Asterisk statistically significant difference (*p* < 0.05) from the corresponding control group.

Cell viability and functions associated with implants are closely related to the physical, chemical, and biological characteristics of the materials used [9,25], which in turn affect *in vivo* efficacy [26]. To elucidate the effects of plasma treatment and surface roughness on osteogenic activities, the biological functions of MG63 cells cultured on samples were evaluated. The two controls exhibited an increased absorbance level of mitochondrial activity with an increasing culture time, as shown in Figure 5. Cells were attached more to the hydrophilic plasma-treated surfaces than corresponding hydrophobic control surfaces at all culture times. A significantly (*p* < 0.05) higher attachment of cells was observed for the treated surfaces than those for the surface of the control after 3 h of culture. Several *in vitro* studies have shown that hydrophilic surfaces have a higher early level of cell attachment than the hydrophobic surface [4,12]. Regarding the effect of the surface roughness, it seems not be dependent on the cell attachment, although the underlying assumption in rough surface designs is that increased surface areas would be more favorable for cell attachment due to mechanical interlocking. Ito *et al.* reported that no clear differences were observed in initial cell attachment on micro- and nano-topographies on the surface of TZP [27]. Similar to the findings, in this study, the Ra values of the used samples were between 30 and 350 nm. In contrast, Yamashita *et al.* found that the number of attached cells on the rough YZP samples (1.01 µm in Ra) was significantly higher than that on the smooth samples (0.18 µm in Ra) [28]. Although an appropriate surface roughness and topography could produce effective mechanical interlocking at the initial cell attachment stage, the further investigation is required to clarify this relationship.

### 2.6. Cell Proliferation

**Figure 6:**
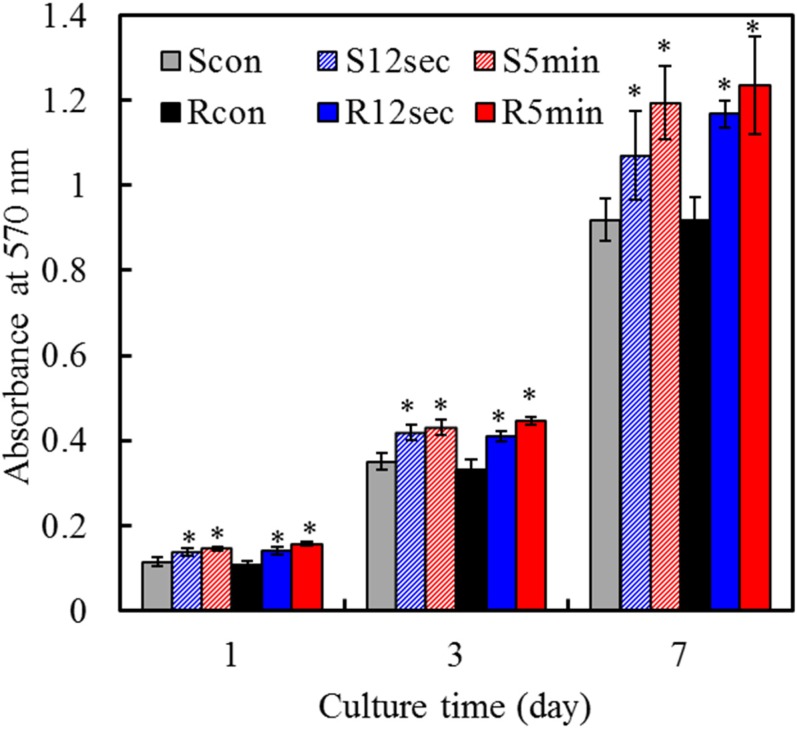
MTT assay for MG63 cells cultured on various samples to reveal cell proliferation at various time points. Asterisk statistically significant difference (*p* < 0.05) from the corresponding control group.

Figure 6 shows that absorbance steadily increased on all samples on days 1–7, which indicates increasing numbers of viable cells. The proliferation of MG63 cells cultured on various plasma-treated surfaces was higher than that on the surfaces of the corresponding controls at all culture time-points. For example, on day 7, the absorbance value for 5-min-treated rough sample (R5min) was significantly (*p* < 0.05) 34% greater than that of the corresponding control (Rcon). It is noticeable that no significant differences (*p* > 0.05) in cell proliferation were detected between smooth and rough groups at any culture time-point.

### 2.7. Cell Differentiation

**Figure 7:**
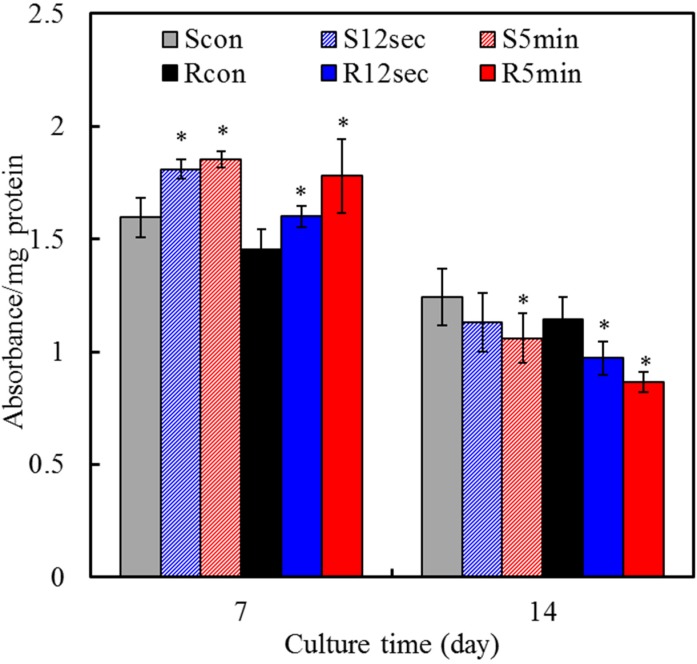
ALP assay on MG63 cells for cell differentiation on various sample surfaces after 7 and 14 days of culture. Asterisk statistically significant difference (*p* < 0.05) from the corresponding control group.

The intracellular ALP level was measured to observe the functional activity of cells. ALP is an early marker for osteoblast differentiation and is produced in high levels during the bone formation phase [29,30]. On day 7, the significant 16% and 22% increments (*p* < 0.05) in the ALP level were measured for smooth and rough groups with 5-min plasma treatment, respectively, compared with the corresponding controls (Figure 7). Of note, the ALP changes of all groups were downregulated after 14 days in culture, and plasma-treated samples occurred more remarkably compared to the control. Secretion of ALP from MG63 cells began earlier during culture on the plasma-treated sample surfaces than on the control surface, in particular for 5 min-treatment. It is generally accepted that an increase in the specific activity of ALP in bone cells reflects a shift to a more differentiated state [31]. ALP enzyme activity is also associated with the initiation of matrix mineralization, and its activity is downregulated after the start of mineralization. According to the literature [20], osteoblastic cells cultured on hydrophilic surfaces produce more differentiation markers represented by the increased ALP specific activity. Cell differentiation studies, in common with results of cell proliferation assay, showed a significant impact of plasma treatment than surface roughness.

### 2.8. Mineralization

**Figure 8:**
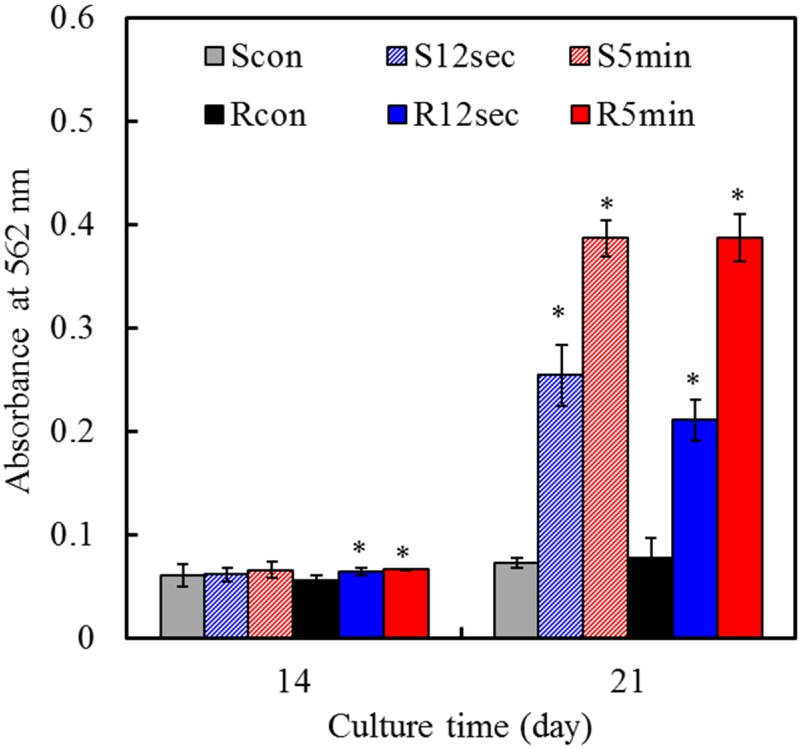
Quantification of calcium mineral deposits by Alizarin Red S assay of MG63 cells cultured on various samples surfaces after culture for 14 and 21 days. Asterisk statistically significant difference (*p* < 0.05) from the corresponding control group.

To more fully assess the role of surface modification in cell functions, mineralization ability was examined. The ability of cells to produce a mineralized matrix and nodules in materials is important for bone regeneration [32]. Alizarin Red S staining is a common histochemical technique used to detect calcium deposits in mineralized tissues and cultures. In the present study, quantification of calcium mineral deposits showed that with increasing culture time, mineral deposition increased for the cells cultured on all samples, as shown in Figure 8. On Day 21, more mineral deposition was found in cells cultured on the 5-min treated samples than the others. These results were in line with results reported by other researchers [33] and supported the hypothesis that the materials with increased hydrophilicity had the increased rate and extent of bone formation.

Another noted fact was that variations in cell responses due to plasma treatment were more significant than those resulting from the surface roughness of the materials examined. This finding, in common with a previous study [34], showed a significant impact of chemical composition, with the emphasis on the superior biological function induced by plasma treatment compared with roughness values ranging from 30 nm to 300 nm in Ra. Dulgar-Tulloch *et al.* documented that hMSC adhesion was dependent upon both the substrate chemistry [hydroxyapatite (HA), titania, and alumina] and grain size, but not on surface roughness or crystal phase [35]. In a word, highly hydrophilic surfaces seem more desirable than hydrophobic ones in view of their interactions with biological fluids, cells and tissues [20,33]. Nevertheless, in addition to the consideration of surface wettability, surface functional groups are another prominent influence on cell response [4,12]. A surface with an organized arrangement of functional groups can act as a site for the protein adsorption and subsequent cell growth. Surface polarity of biomaterials is also recognized as one of the important factors in interacting with the cells. For example, the polarized HA is beneficial for cell adhesion and motility [36]. Therefore, further study is needed to clarify the presences of surface polarity and functional groups of plasma-treated TZP samples and their effect on cell functions. Last but not least, the results of the present study clearly demonstrated that oxygen plasma treatment is an effective method in improving surface properties of the zirconia implants, which can actively support the proliferation and differentiation of osteoblastic cells.

## 3. Experimental Section

### 3.1. Oxygen Plasma Treatment

Commercial Cercon^®^ 3 mol% yttria-stabilized tetragonal zirconia polycrystals (TZP; Dentsply Ceramco, York, PA, USA) with dimensions of 12 mm in diameter and 0.5 mm in thickness were used. Before oxygen plasma treatment, half of the samples were mechanically polished to #1200 grit level, and the other half samples were sandblasted using 50 μm Al_2_O_3_ particles with an application period of 30 s. The oxygen plasma treatment of preteated TZP samples was performed using a plasmochemical generator (Femto, Diener Electronic, Ebhausen, Germany). The chamber was evacuated to a base pressure lower than 0.4 mbar, and then backfilled with high purity oxygen gas until a working pressure of 0.8 mbar was obtained. The plasma was generated using a source power of 200 W and the frequency of 40 kHz for 12 s and 5 min. The sample codes “Scont”, S12 s” and “S5 min” were used to indicate the smooth samples resulted from mechanically polishing and subjected to plasma treatment for 0 s, 12 s and 5 min, respectively. Similarly, the sandblasting-induced rough samples with and without plasma treatment were hereafter designated as “Rcont”, R12 s” and “R5 min”. After finishing the plasma treatment, the assays such as contact angle measurement, XPS analysis and cell culture were performed immediately.

### 3.2. Topography and Composition

The topography and surface roughness of the treated TZP disks were measured using an atomic force microscope (AFM; BRUKER Dimension FastScan^®^, Santa Barbara, CA, USA). The AFM system was operated in air by the tapping mode using a silicon nitride (SiN) cantilever with a sharp tip at its end that was used to scan the disk surface. The images of 40 µm × 40 µm area were obtained with the scan rate of 1 line/s and 256 number of sampling per line. Ra (arithmetic mean roughness) is the root mean square average of the roughness profile ordinates. Root mean square roughness (Rq) is the square root of the sum of the squares of the individual heights and depths from the mean line. The results were obtained from three separate measurements from AFM.

High resolution X-ray diffractometry (HRXRD; Bruker D8 Discover SSS, Karlsruhe, Germany) was used to investigate the phase composition, which was operated at 40 kV and 40 mA at a scanning speed of 1°/min. The chemical composition of various surfaces was measured with an X-ray photoelectron spectroscopy system (XPS; PHI 5000 VersaProbe, ULVAC-PHI Inc., Osaka, Japan) equipped with an Al Kα X-ray source (excitation energy: 1486.6 eV) that had a 21.4-W source energy at the anode.

### 3.3. Estimation of Contact Angle

The static water contact angles were determined by the sessile drop method at room temperature (25 °C, 65% relative humidity). Using a micropipette, a droplet (5 μL) of water was placed on the sample surface. The water contact angle, expressed as the mean ± standard deviation, was calculated from six samples. The variations of the hydrophilicity with various aging time-points (1 h, 1 day, 7 days, 15 days, 30 days and 60 days) in a damp proof box were assessed, which was acted as an index to estimate the stability of surface modification.

### 3.4. Cell Culture

The biocompatibility of the treated samples was evaluated by incubation with MG63 human osteoblast-like cells (BCRC 60279; Bioresources Collection and Research Center, Hsinchu, Taiwan). Specimens were placed in 24-well culture plates after plasma treatment. MG63 cells were added to each well and incubated at 37 °C in a 5% CO_2_ atmosphere. The culture medium was composed of Dulbecco’s modified eagle medium (DMEM; Gibco, Grand Island, NY, USA) supplied with 10% fetal bovine serum (Gibco), 1% penicillin (10,000 U/mL)/streptomycin (10,000 μg/mL) solution (Gibco), 10–8 M dexamethasone (Sigma-Aldrich; St. Louis, MO, USA), 50 μg/mL ascorbic acid (Sigma-Aldrich), and 10 mM β-glycerophosphate (Sigma-Aldrich).

### 3.5. Cell Attachment and Proliferation

To assess the attachment, the cells at a density of 2 × 104 cells/well were cultured for 3, 6 and 12 h. Cell proliferation was performed with an initial cell density of 5 × 103 cells/well on days 1, 3 and 7. After the established incubation period, cell number was examined using the MTT (3-(4,5-dimethylthiazol-2-yl)-2,5-diphenyltetrazolium bromide) assay, in which tetrazolium salt is reduced to formazan crystals by the mitochondrial dehydrogenase of living cells. Briefly, 2 h before the end of the incubation period, 2 mL of MTT solution (2 mg/mL in DMEM containing 1% FBS) and 2 mL of dimethylsulfoxide (DMSO; Sigma-Aldrich) were also added to each well. The plates were then shaken until the formazan crystals had dissolved, and 200 µL of the solution from each well was transferred to a 96-well tissue-culture plate. Plates were read in a Sunrise microplate reader (Tecan Austria Gesellschaft, Salzburg, Austria) at 570 nm, with a reference wavelength of 600 nm. The results were reported in terms of absorbance. The value at absorption maximum of 570 nm can be used to measure the amount of dissolved formazan, which in turn, it indicates the amount of viable cells on the disk surfaces during the culture periods. That is an increase in the amount of MTT formazan formed, which increases in absorbance, due to an increase in viable cell number. The results were obtained in three independent measurements.

### 3.6. Cell Differentiation

To evaluate early cell differentiation, the alkaline phosphatase (ALP) activity assay was performed using a TRACP & ALP assay kit (Takara, Shiga, Japan) according to the manufacturer’s instructions. Briefly, after culture for 7 and 14 days the cells cultured at a density of 5000 cells/well on specimens were washed with 0.9% NaCl and lysed with 100 μL of 1% NP-40 (Sigma-Aldrich) in 0.9% NaCl. For measurement purposes, 90 μL of cellular lysate was mixed with equal volume of the substrate solution (20 mM Tris-buffer, 1 mM MgCl_2_, 12.5 mM p-nitrophenyl phosphate; pH 9.5) and reacted at 37 °C for 1 h. The reaction was stopped by the addition of 45 μL of 0.5 M NaOH and read at 405 nm using a Sunrise Microplate Reader. The ALP activity was normalized to the amount of protein of each specimen, calculated using the BCA protein assay reagent (Bio-Rad, Hercules, CA, USA). ALP activity was expressed as absorbance per mg of protein. The data provided for each group were the mean of three independent specimens.

### 3.7. Mineralization

The mineralized matrix synthesis was analyzed using an Alizarin Red S staining method, which can identify calcium deposits. After culturing for 14 and 21 days, the cells were washed with PBS and fixed in 4% paraformaldehyde (Sigma-Aldrich) for 10 min at 4 °C. This was followed by staining for 10 min in 0.5% Alizarin Red S (Sigma-Aldrich) in PBS at room temperature. The stained cells were completely washed with PBS to reduce nonspecific Alizarin Red S stain and then observed using an optical microscope (BH2-UMA; Olympus, Tokyo, Japan). To quantify matrix mineralization, the calcium mineral precipitate was destained by 10% cetylpyridinium chloride (Sigma-Aldrich) in PBS for 30 min at room temperature. The absorbance of Alizarin Red S extracts was measured at 562 nm using a Sunrise microplate reader. Mean absorbance values were obtained from three independent experiments.

### 3.8. Statistical Analysis

All results are expressed as the mean ± standard derivation for the number of experiments indicated, unless otherwise stated. One-way analysis of variance (ANOVA) was used to evaluate the significance of differences between means. Scheffe’s test was used to determine the significance of the standard deviations in the measurement data of each sample under different experimental conditions. In all cases, results with a *p* value of less than 0.05 were considered statistically significant.

## 4. Conclusions

Strategies for modifying the nature of the material surfaces may affect cell-material interactions, which in turn influence tissue development. The use of oxygen plasma aimed at changing surface characteristics of ZrO_2_ dental ceramic, thereby promoting the biocompatibility. In light of the results obtained in this study, the wettability of ZrO_2_ were noticeably improved by oxygen plasma treatment that was effective for retaining a stable surface hydrophilicity, although the plasma treatment did not change the surface topography. The improved wettability did actively promote the attachment, proliferation and differentiation of human osteoblast-like cells. Overall, these findings consistently demonstrated that the oxygen plasma treatment led to an increase in hydrophilicity and enhanced cell responses.
